# Intrathecal (intraventricular) polymyxin B in the treatment of patients with meningoencephalitis by A*cinetobacter baumanii *and *Pseudomonas aeruginosa*

**DOI:** 10.1186/cc9655

**Published:** 2011-03-11

**Authors:** SK Macedo, IP Gonçalves, GVdO Bispo, LRd Almeida, LBdA Brito

**Affiliations:** 1 São José do Avaí Hospital, Itaperuna, Brazil

## Introduction

Intraventricular therapy (IVT) with polymyxin B (PolyB), an antibiotic with similar pharmacological action to colistin (PolyE), by external ventricular derivation (EVD) has the main goal of offering major bioavailability of the drug, since its use by intravenous and direct action are restricted by the blood-brain barrier, with penetration of only 25%. *Pseudomonas aeruginosa *is a Gram-negative bacterium, multidrug resistant, which has a characteristic of secreting exotoxin A. Along with *Acinetobacter baumannii*, it has expressed a great risk to the lives of patients with meningoencephalitis. The patient of the present report had arterial venous malformations followed by hemorrhagic stroke, which caused elevated intracranial pressure. The objective is to show an example of the effect of IVT PolyB in a patient with meningoencephalitis infection by multidrug-resistant Gram-negative bacteria (*A. baumannii *and *P. aeruginosa*), common in the ICU.

## Methods

A literature review was made on the subject of therapy with PolyB about the pharmacological characteristics, nephrotoxicity and neurotoxicity. A comparative table of the profile of resistance of the strain treated in this study was created, with the intrinsic resistance of the species. Also, the development of liquor evolution (culture and routine) of the patient before the treatment was monitored, until negative liquor. We analyzed the life and effectiveness of EVD, the colonizer germ and monitoring of the serial aspect of the liquor.

## Results

The patient was treated with intravenous and intrathecal administration of PolyB (IVT) between 14 November and 28 November 2008. On 14 November 2008, therapy was started with PolyB intravenous administration of 1,500,000 UI (20,000 UI/kg/day) once a day, on every day of treatment, and IVT by EVD: 50,000 UI in solution once a day during the first 3 days, and on alternate days for all of the treatment. As a result of the use of intrathecal PolyB intravenously, effectiveness was proven in the routines of liquor negative for such germs, showing no reports of neurotoxicity and nephrotoxicity.

## Conclusions

IVT PolyB proved to be very efficient, treating this meningo-encephalitis quickly. No toxic effect was associated with the drug.
